# miR-375-3p negatively regulates osteogenesis by targeting and decreasing the expression levels of LRP5 and β-catenin

**DOI:** 10.1371/journal.pone.0171281

**Published:** 2017-02-03

**Authors:** Tianhao Sun, Chen-Tian Li, Lifeng Xiong, Ziyu Ning, Frankie Leung, Songlin Peng, William W. Lu

**Affiliations:** 1 Department of Orthopaedics and Traumatology, Li Ka Shing Faculty of Medicine, The University of Hong Kong, Hong Kong SAR, China; 2 Department of Spine Surgery, Shenzhen People's Hospital, Jinan University Second College of Medicine, Shenzhen, China; 3 Department of Microbiology, Li Ka Shing Faculty of Medicine, The University of Hong Kong, Hong Kong SAR, China; 4 Department of Medicine, Li Ka Shing Faculty of Medicine, The University of Hong Kong, Hong Kong SAR, China; Van Andel Institute, UNITED STATES

## Abstract

Wnt signaling pathways are essential for bone formation. Previous studies showed that Wnt signaling pathways were regulated by miR-375. Thus, we aim to explore whether miR-375 could affect osteogenesis. In the present study, we investigated the roles of miR-375 and its downstream targets. Firstly, we revealed that miR-375-3p negatively modulated osteogenesis by suppressing positive regulators of osteogenesis and promoting negative regulators of osteogenesis. In addition, the results of TUNEL cell apoptosis assay showed that miR-375-3p induced MC3T3-E1 cell apoptosis. Secondly, miR-375-3p targeted low-density lipoprotein receptor-related protein 5 (LRP5), a co-receptor of the Wnt signaling pathways, and β-catenin as determined by luciferase activity assay, and it decreased the expression levels of LRP5 and β-catenin. Thirdly, the decline of protein levels of β-catenin was determined by immunocytochemistry and immunofluorescence. Finally, silence of LRP5 in osteoblast precursor cells resulted in diminished cell viability and cell proliferation as detected by WST-1-based colorimetric assay. Additionally, all the parameters including the relative bone volume from μCT measurement suggested that LRP5 knockout in mice resulted in a looser and worse-connected trabeculae. The mRNA levels of important negative modulators relating to osteogenesis increased after the functions of LRP5 were blocked in mice. Last but not least, the expression levels of LRP5 increased during the osteogenesis of MC3T3-E1, while the levels of β-catenin decreased in bone tissues from osteoporotic patients with vertebral compression fractures. In conclusion, we revealed miR-375-3p negatively regulated osteogenesis by targeting LRP5 and β-catenin. In addition, loss of functions of LRP5 damaged bone formation in vivo. Clinically, miR-375-3p and its targets might be used as diagnostic biomarkers for osteoporosis and might be also as novel therapeutic agents in osteoporosis treatment. The relevant products of miR-375-3p might be developed into molecular drugs in the future. These molecules could be used in translational medicine.

## Introduction

Osteoporosis is a global and severe public health issue, which affects one-third women and one-fifth men in the worldwide [1].The pathological mechanisms of osteoporosis are not clearly understood. Therefore, it is essential to uncover new molecules that might play significant roles in the disturbed osteogenesis, which is a key process responsible for the pathogenesis of osteoporosis.

MicroRNAs (miRNAs) are non-coding RNA molecules containing approximate 20 nucleotides. miRNAs play important roles in many diseases including some musculoskeletal disorders, such as osteoporosis. They can also be used as the biomarkers and potential therapeutical targets. Substantial biological processes such as cell proliferation, differentiation and apoptosis are regulated by miRNAs and genes. For instance, our recent study showed that miR-9-5p, miR-675-5p and miR-138-5p damaged skeletal cell proliferation and differentiation [2]. miRNAs decrease the expression of genes by binding to the 3′-untranslated region (3′UTR) of target mRNAs.

The balance between proliferation and apoptosis of bone cells decides the size of osteoblast populations [3]. Understanding the mechanisms of skeletal cell proliferation and cell apoptosis will help to study the osteogenesis and develop better treatments for osteoporosis. MC3T3-E1 is an osteoblast precursor cell line derived from mouse. The cells are commonly used as model systems in skeletal molecular biology. MC3T3-E1 is one of the most physiologically relevant systems for investigation of osteoblasts.

There are many genes and their coded proteins regulating osteogenesis and bone formation. Some of them play positive roles in osteogenesis whilst the others had negative effects. Thus, they could be used as the biomarkers and indicators of osteogenesis. Runt-related transcription factor 2 (RUNX2) is an important transcription factor in osteoblast differentiation, so it is a vital biomarker for active bone formation. Deficiency of sclerostin (SOST) increases bone formation [4]. Mothers against decapentaplegic homolog 7 (SMAD7) binds to Pellino-1 and then inhibits NF-κB activity. SMAD7 blocks the differentiation of osteoblastic cells [5]. All the aforementioned molecules could be used as the indicator of osteogenesis.

Wnt signaling pathways play vital roles in the osteogenesis and bone formation [6–10]. For instance, WNT7B promoted bone formation because induction of WNT7B in the osteoblast lineage significantly enhanced bone mass due to increased osteoblast activity [10]. Glycogen synthase kinase 3 (GSK3) and casein kinase 1α (CK1α) belong to the destruction complex of Wnt signaling pathways. The destruction complex degrades β-catenin. Several previous studies showed that Wnt signaling pathways were regulated by miR-375 [11,12,13]. Therefore, in the present study we explored whether miR-375-3p could affect osteogenesis. LRP5 is an important co-receptor of the Wnt signaling pathways [14, 15]. Our previous studies showed that LRP5 played vital roles in osteogenesis in vitro [2]. Though the roles of Wnt signaling pathways in osteogenesis have been studied, the studies of the exact roles and effects of LRP5 in vivo are few and needed.

In the present study, we investigated the roles of miR-375-3p in osteogenesis and verified the binding relationship between the miR-375-3p and its targets LRP5 and β-catenin. Then we further studied the functions of LRP5 in vivo.

## Results

### miR-375-3p damaged osteogenesis by inducing cell apoptosis

**Fig 1:**
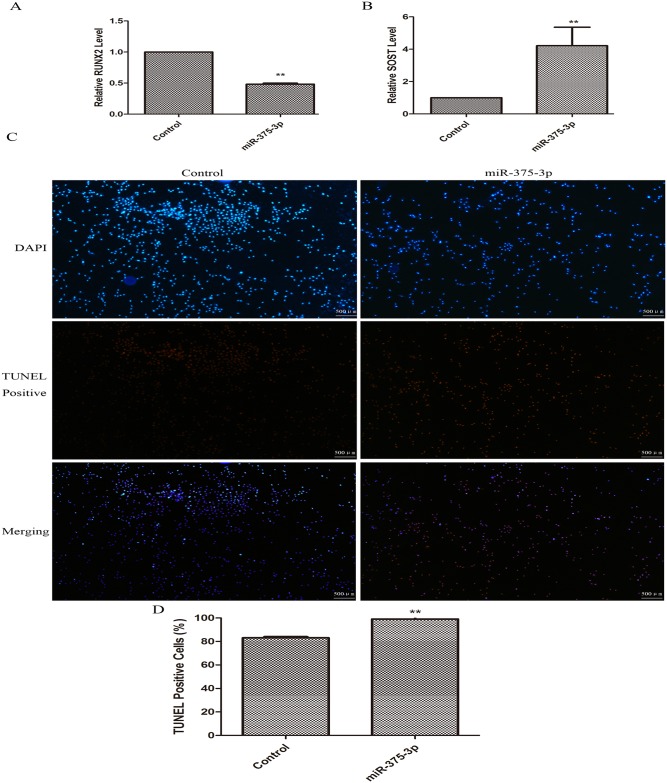
miR-375-3p damaged osteogenesis by inducing cell apoptosis. (A) Relative expression levels of RUNX2 in MC3T3-E1 cells treated with miR-375-3p mimics. (B) Relative expression levels of SOST in MC3T3-E1 cells treated with miR-375-3p mimics. (C) Representative TUNEL staining images of MC3T3-E1 cells. More TUNEL positive staining cells (Red) were found after the cells were transfected with miR-375-3p mimics. (D) Quantification of TUNEL-positive cells. * p < 0.05, ** p < 0.01. n = 3/group.

Our results showed that the expression levels of osteoblast differentiation biomarker RUNX2 declined (Fig 1A) while the negative regulators (SOST) of osteogenesis rose sharply (Fig 1B) after the osteoblast precursor cells MC3T3-E1 were transfected with miR-375-3p mimics (2.5 nM), suggesting that miR-375-3p damaged osteogenesis. We then hypothesized that the reasons why miR-375-3p damaged osteogenesis were that it induced cell apoptosis. To verify this point, we conducted TUNEL cell apoptosis assay. Fig 1C and 1D showed that the percentage of the red (TUNEL positive) cells was much higher in the miR-375-3p mimics treated group than the control group, suggesting that miR-375-3p induced cell apoptosis in MC3T3-E1 cells. Taken together, all these results strongly suggested that miR-375-3p negatively regulated osteogenesis.

### miR-375-3p targeted LRP5 and β-catenin

**Fig 2:**
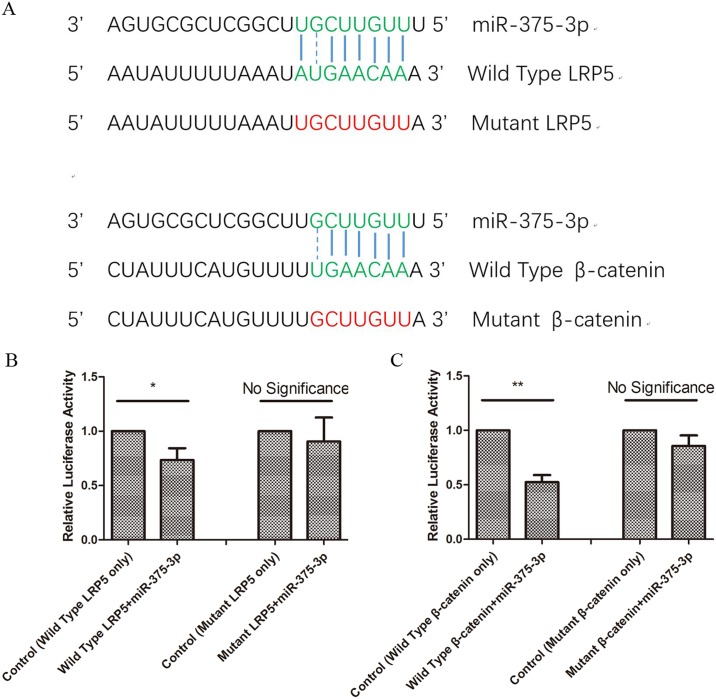
miR-375-3p targeted LRP5 and β-catenin. (A) The binding sites between miR-375-3p and the 3’UTR of LRP5 and β-catenin. (B) The relative luciferase activity of MC3T3-E1 cells transfected with luciferase-wild type (or mutant) LRP5 plasmids and miR-375-3p mimics. (C) The relative luciferase activity of MC3T3-E1 cells transfected with luciferase-wild type (or mutant) β-catenin plasmids and miR-375-3p mimics. * p < 0.05. ** p < 0.01, NS, not significant. n = 3/group.

We further studied the downstream targets of miR-375-3p. We used the prediction tool miRanda-mirSVR-cBio@MSKCC (http://www.microrna.org/microrna/home.do), and LRP5 and β-catenin were predicted to be the targets of miR-375-3p. Fig 2A showed the binding sites between miR-375-3p and the 3’UTR of LRP5 or β-catenin. Then we constructed four plasmids, namely wild type LRP5, mutant LRP5, wild type β-catenin and mutant β-catenin. Transfection of miR-375-3p mimics resulted in a significant reduction in luciferase activity in MC3T3-E1 cells transfected with wild type plasmids (Fig 2B and 2C), while the inhibition of luciferase activity by miR-375-3p was abrogated in cells transfected with mutant plasmids (Fig 2B and 2C). In other words, miR-375-3p inhibited its wild type targets more significantly than mutant ones, so resistance of mutant luciferase vectors to miRNAs confirmed the specific targeting. Taken together, these results suggested that miR-375-3p directly targeted LRP5 and β-catenin in MC3T3-E1 cells.

### miR-375-3p arrested the expression levels of LRP5 and β-catenin

**Fig 3:**
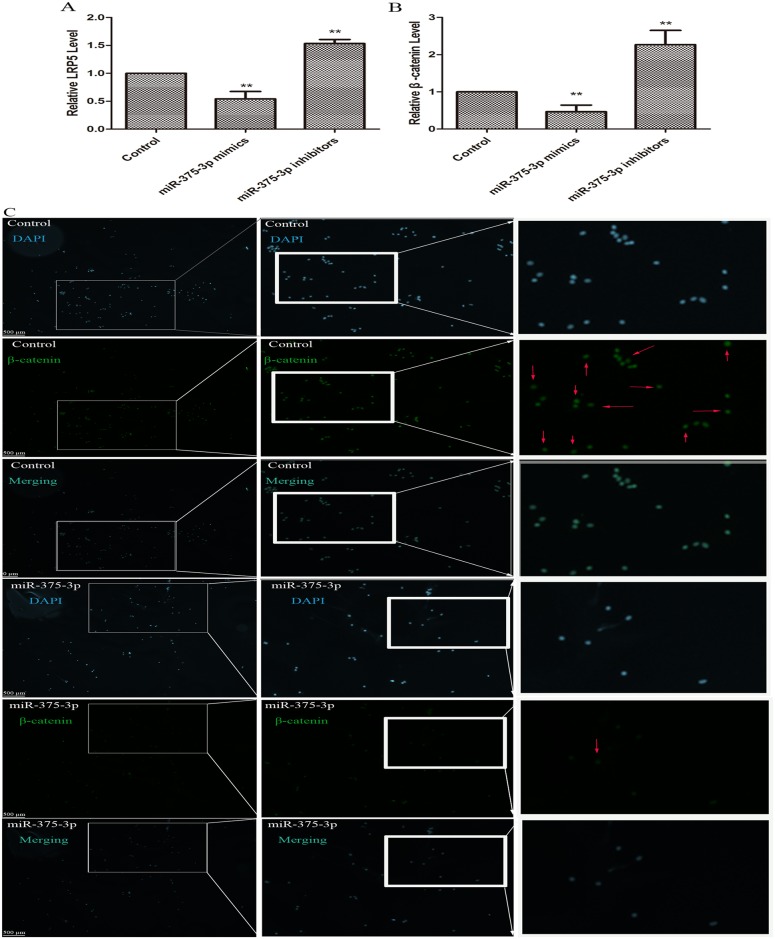
miR-375-3p arrested the expression of LRP5 and β-catenin. (A) Relative expression levels of LRP5 in MC3T3-E1 cells upon transfection of miR-375-3p mimics or inhibitors. (B) Relative expression levels of β-catenin in MC3T3-E1 cells upon transfection of miR-375-3p mimics or inhibitors. (C) Representative staining images of MC3T3-E1 cells. Less green cells were found after the cells were transfected with miR-375-3p mimics. * p < 0.05. ** p < 0.01. n = 3/group.

We then transfected the miR-375-3p mimics or inhibitors into the MC3T3-E1 cells and results showed that both LRP5 and β-catenin were downregulated by miR-375-3p mimics while they were upregulated significantly by miR-375-3p inhibitors (Fig 3A and 3B). In other words, miR-375-3p mimics decreased the mRNA levels of LRP5 and β-catenin whilst its inhibitor increased their levels, suggesting that β-catenin, the downstream molecule and effector of LRP5 in the Wnt signaling pathways, was also suppressed by miR-375-3p.

To further confirm β-catenin was restrained by miR-375-3p at protein level, we conducted immunocytochemistry, and results showed the number of green cells and the degree of green were lower after the cells were treated with miR-375-3p mimics (Fig 3C), indicating that the protein levels of β-catenin were decreased by miR-375-3p. Taken together, these data strongly suggested that the miR-375-3p downregulated β-catenin at both mRNA and protein level.

### Loss of function of LRP5 resulted in damaged osteogenesis in vitro and in vivo

**Fig 4:**
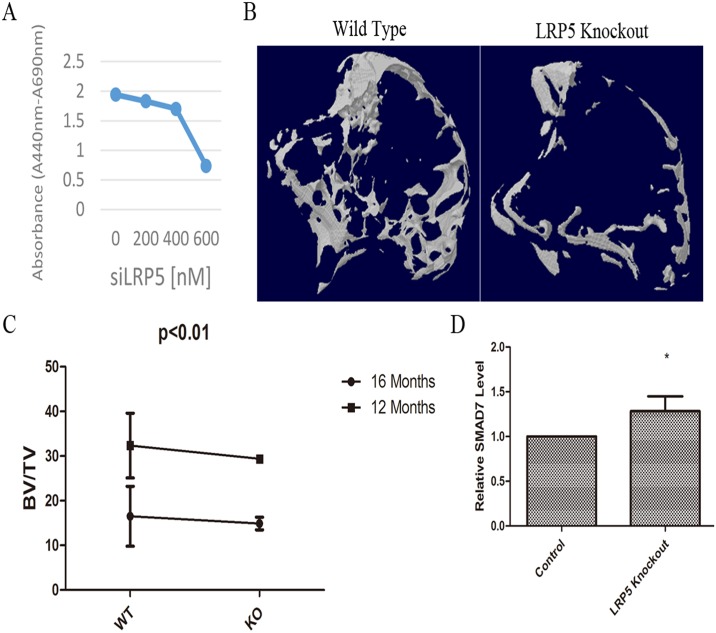
Loss of function of LRP5 resulted in damaged osteogenesis in vitro and in vivo. (A) The relative absorbance of MC3T3-E1 cells transfected with a serial dilution of siLRP5 in the WST-1-based colorimetric assay. (B) Representative 3D images of bone microstructure in the knee-joints in LRP5 WT and KO mice. (C) Quantification of trabecular bone volume fraction (BV/TV) in the knee-joints in WT or KO mice was determined from the μCT measurement. (D) Relative expression levels of SMAD7 in the bone tissues of mice. * p < 0.05, ** p < 0.01, n = 3/group.

According to our results, miR-375-3p was a negative regulator of osteogenesis and it decreased the levels of LRP5. We then silenced the functions of LRP5 to imitate the effects of miR-375-3p. We firstly explored the roles of LRP5 on osteogenesis in vitro. The results of WST-1-based colorimetric assay showed that MC3T3-E1 cell viability and cell proliferation rates decreased after silence of LRP5 in a dose-dependent manner (Fig 4A), indicating that LRP5 was essential for skeletal cell viability. The results suggested that loss function of LRP5 in osteoblastic cells led to decreased cell viability.

Since our results showed that silence of function of LRP5 led to impaired osteogenesis in vitro, we then tried to verify this point in vivo by using LRP5 knockout (KO) mice. As showed in Fig 4B, LRP5 KO resulted in a looser and worse-connected trabeculae. Quantification analysis demonstrated that the relative bone volume (BV/TV) was significantly (p < 0.01) lower in LRP5 KO mice than in wildtype (WT) mice (Fig 4C). Taken together, all these data suggested that loss of function of LRP5 in vivo resulted in decreased bone volume. To further confirm the positive effects of LRP5 on bone formation, we checked whether LRP5 KO in vivo resulted in increased expression of negative regulators (SMAD7) of osteogenesis. Results showed that loss of function of LRP5 in vivo led to significant increase of SMAD7 (Fig 4D).

### The expression dynamics of effectors of miR-375-3p during osteogenesis

**Fig 5:**
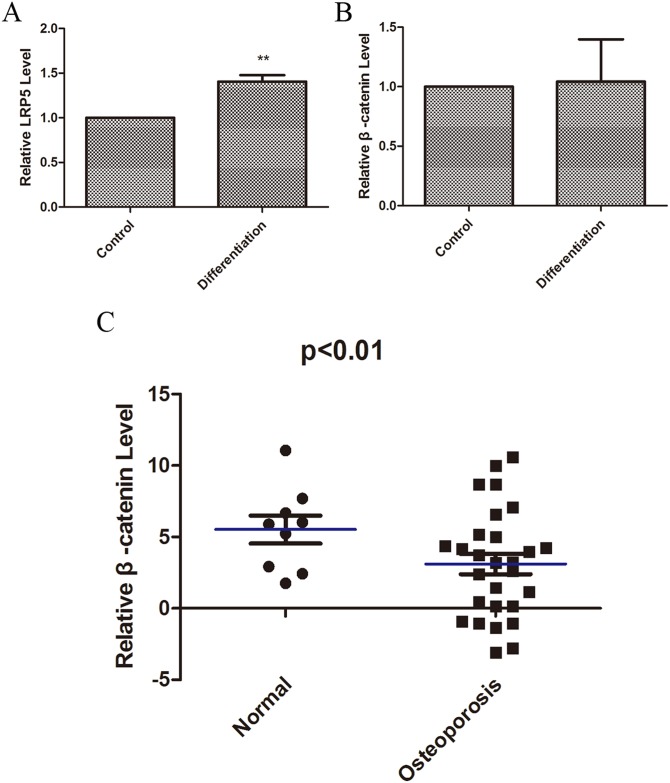
The expression dynamics of effectors of miR-375-3p during osteogenesis. (A) Relative expression levels of LRP5 during MC3T3-E1 differentiation and osteogenesis. (B) Relative expression levels of β-catenin during MC3T3-E1 differentiation. (C) Relative expression levels of β-catenin in bone tissues from osteoporotic patients and their controls. * p < 0.05. ** p < 0.01.

We further examined the expression levels of the downstream effectors and targets of miR-375-3p (LRP5 and β-catenin) during osteogenesis of MC3T3-E1. During differentiation and osteogenesis of MC3T3-E1, the expression levels of LRP5 and β-catenin increased (Fig 5A and 5B), especially LRP5 (Fig 5A), indicating that LRP5 and β-catenin might play vital positive roles in osteoblastic differentiation.

Finally, we tried to have a better understanding of the important downstream effective molecules of miR-375-3p (β-catenin) expression pattern in bone disease. First of all, we selected osteoporosis and osteoporotic fracture as the bone disease, because one of the most important causes of osteoporosis was that abnormal and disordered osteogenesis and damaged osteoblastic differentiation [16–18]. In our present study we explored the roles of β-catenin targeted by miR-375-3p in osteoblastic differentiation cell model MC3T3-E1. Thus, we analyzed the levels of β-catenin in bone tissues from twenty-eight osteoporotic patients with vertebral compression fractures and nine age-matched healthy controls. Results showed that the levels of β-catenin in human bone tissues from patients with osteoporosis significantly decreased (Fig 5C), indicating that osteoporosis was closely related to declining β-catenin.

## Discussion

**Fig 6:**
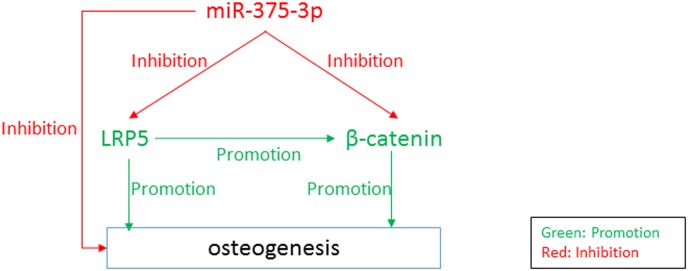
The flowchart of the main study factors and their relationships.

The current study explored the effects of miR-375-3p and LRP5 on osteogenesis. We revealed that miR-375-3p negatively affected osteogenesis. Further studies showed that miR-375-3p decreased the levels of LRP5 and β-catenin by directly binding to their 3’UTR. Finally, we revealed loss function of LRP5 impaired osteogenesis and bone formation in vivo. The main factors and their relationships were showed in the Fig 6. This study provided data which were useful for diagnosis and treatment of osteoporosis.

In this study we revealed that miR-375-3p targeted both LRP5 and β-catenin and significantly decreased the expression of β-catenin, indicating that miR-375-3p might strongly suppress Wnt signaling pathways, which were essential for bone formation. Further studies including the animal studies of the functions of miR-375-3p are needed. miR-214 was reported to significantly inhibit bone formation [19]. In fact, we found that miR-214 could also target both LRP5 and β-catenin, which might be the crucial reason why miR-214 strongly suppressed osteogenesis. Because miR-375-3p has these effects which are similar to miR-214, it might be a promising miRNAs inhibiting bone formation.

Mi-375 served as a tumor suppressor in gastric cancer [20] and in laryngeal squamous carcinoma [21]. Overexpression of miR-375 also induced G1 cell cycle arrest through a decrease in the expression of cyclin D1 and cyclin D3, which was consistent with our data. In addition, another study also showed that miR-375 increased apoptosis [22]. Most studies of miR-375 focused on the cancer including the osteosarcoma. Most of these studies showed that miR-375 negatively regulated carcinogenesis [23,24]. The studies of the effects of miR-375 on osteogenesis and bone formation are few. To our knowledge, there are no studies of the effects of miR-375-3p on osteogenesis. In our study we revealed that miR-375 might negatively regulate osteogenesis by inducing cell apoptosis, and further studies of miR-375 should be conducted.

MiRNAs bind to their target genes and degrade them. We revealed that miR-375-3p specifically targeted to the LRP5 and β-catenin. Decreased protein levels of LRP5 and β-catenin by Western Blot could be verified to strengthen the statements. Based on our consistent findings from in vitro studies, it is reasonable to speculate that the blocking of function of miR-375-3p, such as by injection of miR-375-3p antagomir, Wnt/LRP5 signaling can be activated, and therefore the bone loss due to the decreased bone formation in aged mice might be rescued or even reversed.

Wnt signaling pathways play vital roles in the skeletal cell proliferation and bone formation. However, the exact mechanisms are not very clear. Because LRP5 is an important component in the Wnt signaling pathways, we studied its effects. We revealed that it was LRP5 in the Wnt signaling pathways that exerted very important positive effects in the skeletal cell proliferation and bone formation.

Kato et al. [25] found that LRP5 KO mice had low bone mass by using histological analysis and traditional radiographic analysis. In the present study we used μCT and analyzed the important parameter (BV/TV) of bone. Cui et al. [26] stated that loss of LRP5 in osteocytes decreased bone mass. Our studies showed that loss of LRP5 in osteoblast precursor cell (MC3T3-E1) decreased osteogenesis. Yadav et al. [27] investigated the levels of genes affecting cell proliferation, differentiation, bone matrix deposition in the bones of LRP5 KO mice. They found cell proliferation regulator CYCD1 was decreased in LRP5−/− bones. Osteoblast proliferation was lower in the cells from LRP5 KO mice compared to the wild type littermates [28]. In addition, apoptosis was higher in osteoblast-like cells from LRP5 KO mice than in those from wild type mice. Taken together, all these results indicated that LRP5 was essential for bone formation.

In the present study, we revealed that the levels of β-catenin in bone tissues obtained from osteoporotic patients with vertebral compression fractures decreased significantly, indicating that the osteoporotic fractures might be caused by decreased β-catenin. Other studies also showed that β-catenin was essential for bone formation [29]. We also showed that LRP5 and β-catenin were targeted and downregulated by miR-375-3p, indicating that miR-375-3p might strongly downregulate Wnt signaling pathways and bone formation. Thus, studying miR-375-3p in the first place was reasonable.

Clinically, miR-375-3p and its targets LRP5 and β-catenin might be used as diagnostic biomarkers for osteoporosis, osteoarthritis or osteosarcoma because they regulate or disturb osteogenesis. For example, medical workers including doctors and nurses could detect the expression levels of miR-375-3p in the patients’ blood or bone tissues. Our data showed that miR-375-3p negatively modulate osteogenesis, so the expression levels of this miRNA in the specimens from patients with osteoporosis were expected to be upregulated. Increased levels of miR-375-3p in the patients’ bone damaged osteogenesis and bone formation, resulting in osteoporosis. The LRP5 and β-catenin might be as therapeutic targets in osteoporosis treatment. Specifically, we might develop drugs to activate and enhance the functions of LRP5 and β-catenin to treat osteoporosis. miR-375-3p might be also as novel therapeutic agents in osteoporosis treatment. Actually, miRNAs have been investigated their suitability for clinics. For instance, Gallo et al. [30] assessed the pharmacokinetic properties of LNA-anti-miR-221 in mice and monkeys. They found that LNA-anti-miR-221 had optimal tissue bioavailability minimal urine excretion and was measureable in mice vital organs after treatment. Such kinds of studies enlighten the road of miRNAs for clinical use. miR-375-3p, LRP5 and β-catenin as molecules could be used in translational medicine, which means "laboratory-to-clinic". The relevant products of miR-375-3p might be developed into molecular drugs in the future.

## Materials and methods

### Cell culture and transfection

Cell culture of MC3T3-E1 was described in our previous study [2]. MC3T3-E1 differentiation and osteogenesis were induced by supplementing the growth medium with 10 mM β-Glycerophosphate disodium salt (Sigma) and 50 μg/ml L-Ascorbic acid (Sigma). Three days after the treatment, RNA extraction was conducted. For miRNA mimics, miRNA inhibitors and siRNA transfection, we used 0.5% HiPerfect (Qiagen). For luciferase reporter assay, cells were co-transfected with plasmids and miRNA mimics in the presence of Lipofectamine 2000.

### RNA extraction, reverse transcription and qPCR

TRIzol was used for RNA extraction. For expression of gene detection, the RNA was mixed with random reverse transcription primer. The sequences of primers were listed in the Table 1.

**Table 1:** Sequences of all primers.

Primer Name	Sequences (5’–3’)
*Gapdh* (mus musculus) Forward	ATTGTCAGCAATGCATCCTG
*Gapdh* (mus musculus) Reverse	ATGGACTGTGGTCATGAGCC
*Runx2* (mus musculus) Forward	AAGTGCGGTGCAAACTTTCT
*Runx2* (mus musculus) Reverse	ACGCCATAGTCCCTCCTTTT
*Hey1* (mus musculus) Forward	ATCTCAACAACTACGCATCCCAGC
*Hey1* (mus musculus) Reverse	GTGTGGGTGATGTCCGAAGG
*Sost* (mus musculus) Forward	GGAATGATGCCACAGAGGTCAT
*Sost* (mus musculus) Reverse	CCCGGTTCATGGTCTGGTT
*Akt1* (mus musculus) Forward	GGCGAGCTGTTCTTCCACCT
*Akt1* (mus musculus) Reverse	ATTGTCCTCCAGCACCTCGG
*Hes1* (mus musculus) Forward	AAAGACGGCCTCTGAGCAC
*Hes1* (mus musculus) Reverse	GGTGCTTCACAGTCATTTCCA
*Lrp5* (mus musculus) Forward	TACTTTGTCTGCCAGCGTGT
*Lrp5* (mus musculus) Reverse	GGGGCTCCACCAACATACTC
*β-catenin* Forward	TCTGAGGACAAGCCACAAGATTACA
*β-catenin* Reverse	TGGGCACCAATATCAAGTCCAA
*Smad7* (mus musculus) Forward	GGACGCTGTTGGTACACAAG
*Smad7* (mus musculus) Reverse	GCTGCATAAACTCGTGGTCATTG

### Cell proliferation assay

Colorimetric assay (WST-1 based) was used to test cell proliferation. The detailed procedures were described in our previous studies [2].

### TUNEL cell apoptosis assay

ApopTag^®^ Red In Situ Apoptosis Detection Kit (Merck Millipore Corporation) was used in cell apoptosis assay. It detected apoptosis by indirect TUNEL method, utilizing an anti-digoxigenin antibody with a rhodamine fluorochrome. In brief, the cells were seeded into the 12 well chamber (ibidi, Martinsried, Germany) and treated. It was fixed in 1% paraformaldehyde and post-fixed in ethanol: acetic acid (2:1), sequentially applied with Equilibration Buffer, TdT Enzyme, Stop/Wash Buffer, Anti-Digoxigenin Conjugate and Rhodamine/DAPI Staining. When taking photos, we assured consistent parameters of the fluorescence microscopy so that all the pictures were comparable.

### Plasmid construction and luciferase reporter assays

First of all, we designed sequences including the two sequences of LRP5 and two sequences of β-catenin in the Fig 2A. The sequences were synthesized as the oligonucleotides. Then the forward and reverse ones were made into double strand DNA fragments. Then we ligated the four DNA fragments into the pmirGLO Dual-Luciferase miRNA Target Expression Vector (Promega) digested with SacI and XhoI. Colony PCR, agarose gel electrophoresis and sequencing confirmed the ligation was successful. So we successfully inserted the wild type and mutant fragments of the 3’UTR of LRP5 containing the binding sites of miR-375-3p into the luciferase vector. We co-transfected the MC3T3-E1 cells with the plasmids and miR-375-3p mimics. We used Dual-Glo^®^ Luciferase Assay System (Promega) for measuring firefly luciferase activities 48 h after transfection. In brief, we measured firefly luciferase activity by adding Dual-Glo^®^ Luciferase Reagent into the cell medium. The firefly luminescence was measured by the luminometer called VICTOR^™^ X3 Multilabel Plate Reader (PerkinElmer,Waltham, MA, USA). Then we measured Renilla luciferase activity by adding Dual-Glo^®^ Stop & Glo Reagent into the original culture medium. Renilla luminescence was measured in the same plate order as the firefly luminescence was measured. Then we calculated the ratio of luminescence from the experimental reporter (firefly) to luminescence from the control reporter (Renilla), normalized this ratio to the ratio of control wells.

### Immunocytochemistry (ICC) and Immunofluorescence (IF)

The expression levels of β-catenin were detected by ICC and IF. In brief, the MC3T3-E1 cells were seeded in 12 well chamber (ibidi, Martinsried, Germany) and transfected with miR-375-3p. The cells were fixed with 4% paraformaldehyde in PBS, permeabilized with 0.1% Triton X-100, blocked with 10% serum of goat, incubated with primary antibody β-catenin Antibody (Abcam) overnight, and then secondary antibody Goat Anti-Rabbit Ig G (Alexa Fluor 488) (Abcam).

### Animal

LRP5 knockout mice were described previously [2]. All animal procedures were approved by the Committee on the Use of Live Animals in Teaching and Research of The University of Hong Kong (CULATR No. 3500–14). For genotypes analysis of the mice, DNA extraction, PCR and gel electrophoresis were used. To avoid the influences of age and sex on the results, we divided the mice into two subgroups according to their age and sex. In each subgroup, the age and sex are consistent. For example, we have six knee-joints in subgroup 1, including four wild type ones and two knockout ones, and their age and sex are same. The details of these mice were listed in Table 2. All the mice were anesthetized before μCT detection.

**Table 2:** The baseline of mice.

Group	Number	
	Subgroup 1 (Age: 12 months, Sex: M)	Subgroup 2 (Age: 16 months, Sex: F)
Wild Type (control group)	2	2
Knockout (experimental group)	1	1

### μCT measurement

**Fig 7:**
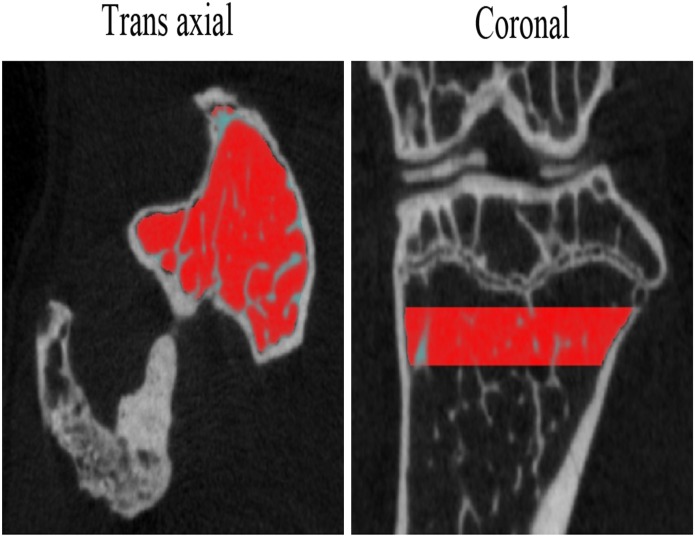
The examples of region of interests.

Trabecular bone microarchitecture of twelve knee-joints of six mice was analyzed with μCT (SkyScan 1076, Kontich, Belgium). All the samples were analyzed by the μCT scanning with the following parameters: 8.665 um isotropic voxel size, 48 kV voltage, 179 mA current, and 1800ms exposure time. The region of interests (ROI) was the trabecular bone area selected manually slice by slice at the region about 0.1mm distal to the end of tibia bone growth plate, and the thickness of ROI was 0.5mm (60 slices) extended to distal side. The examples of ROI were shown in Fig 7. Model-independent 3D measurement methods were used to calculate the trabecular bone volume fraction (BV/TV).

### Patients and controls

The bone tissues were obtained from twenty-eight osteoporotic patients with vertebral compression fractures and nine age-matched healthy controls in the Department of Spine Surgery, Shenzhen People's Hospital (Shenzhen, China). All participants gave written informed consent on the use of clinical specimens for medical research. The samples used in this study were approved by the Committees for Ethical Review of research involving human subjects at the Shenzhen People's Hospital.

### Statistical analysis

Statistical analysis was performed by a t-test or paired t-test, and p<0.05 was considered statistically significant. Mann-Whitney U Test was used for analyzing clinical samples. The software GraphPad Prism was used to draw the charts.

## Conclusions

In conclusion, our findings reveal miR-375-3p inhibits LRP5 and β-catenin mediated osteogenesis by targeting them, and loss function of LRP5 damages osteogenesis.
